# Genome Sequence of Hardyhisp2, a Gammapleolipovirus Infecting Haloarcula hispanica

**DOI:** 10.1128/MRA.00226-21

**Published:** 2021-05-13

**Authors:** Mike Dyall-Smith, Friedhelm Pfeiffer, Pei-Wen Chiang, Sen-Lin Tang

**Affiliations:** a Computational Biology Group, Max Planck Institute of Biochemistry, Martinsried, Germany; b Veterinary Biosciences, Faculty of Veterinary and Agricultural Sciences, University of Melbourne, Parkville, Australia; c Biodiversity Research Center, Academia Sinica, Taipei, Taiwan; Portland State University

## Abstract

Hardyhisp2 virus infects the halophilic archaeon Haloarcula hispanica DSM 4426^T^ and is closely related to His2 (*Pleolipoviridae* family). The viral genome is 16,133 bp long, with terminal inverted repeats of 599 bp. The predicted spike protein is only weakly similar (32% amino acid identity) to that of His2.

## ANNOUNCEMENT

Hardyhisp2 is a novel halovirus that is related to the gammapleolipovirus His2 (1) and was recovered in 1998 from a water sample from the hypersaline Lake Hardy, Victoria, Australia (35°3′58.27″S, 141°44′12.50″E). Originally labeled HH2, it produced turbid plaques of 0.5 to 1 mm (2 to 3 days at 37°C) in soft-layer overlay lawns of the isolating host Haloarcula hispanica DSM 4426^T^. The isolation and plaque purification followed the methods described for halovirus His2 (1). A stock of this virus that had been stored at −80°C since 1999 was thawed, and DNA was extracted using the cetyltrimethylammonium bromide (CTAB)/phenol-chloroform method (2). After the quality and quantity were checked by agarose gel electrophoresis, total DNA was amplified using the Qiagen REPLI-g minikit; the products were checked for suitable size by agarose gel electrophoresis and then purified using QIAamp DNA minikits (Qiagen, Inc., Valencia, CA, USA). One microgram of DNA was sent to the Yourgene Health Co. (Taipei, Taiwan) for sequencing using the Illumina HiSeq 2500 platform (2 × 250-bp paired-end reads). For library construction, DNA was fragmented by sonication, end repaired, and A tailed (Klenow fragment), indexed adapters were added (DNA ligase), and PCR amplification (10 cycles) was performed to enrich for adapter-modified DNA. Sequencing returned 102,128 paired-end Illumina reads (confidence mean, Q36.8; reads with a score of at least Q30, 95.6%) that were quality trimmed (quality trimming criteria, minimum length of 35 bp and error probability of <0.05), resulting in 102,049 reads (average length, 248.6 bp). These reads were assembled using the *de novo* assembler tool within Geneious version 10.2.6 (3). This and other software tools were run with default parameters. A 16,133-bp linear, double-stranded DNA (dsDNA) contig was assembled (521-fold coverage) with an inverted terminal repeat (ITR) of 599 bp. The ITR was very similar to the 525-bp ITR of halovirus His2, with the final 130 bp being 91% identical and the terminal 23 bp being completely identical. The average G+C content was 39.8%, considerably less than that of the host (62.7%) (4). The Hardyhisp2 genome is similar in size to that of His2 (16,067 bp) (1) and shares 82.3% average nucleotide identity (5). The His2 genome carries terminal proteins, but this was not determined for Hardyhisp2. The two viruses probably represent distinct species within the *Gammapleolipovirus* genus (6, 7). Annotation used gene prediction by GeneMarkS-2 (8) and BLAST searches (9) of the NCBI nucleotide/nonredundant databases and predicted 33 coding sequences (CDSs), with most being similar in sequence and gene arrangement to those of His2. The conserved block of pleolipovirus genes specifying structural proteins and a putative NTPase (6) (HdyHp2_120 to HdyHp2_155) includes the gene for the major spike protein (HdyHp2_135), a virion surface protein probably involved in host cell attachment and membrane fusion (10). The spike proteins of Hardyhisp2 and His2 (His2V_gp29) share only 32% amino acid identity, whereas the proteins specified by nearby genes share ≥85% amino acid similarity, suggesting that the two viruses could use distinct binding sites on the cell surface of H. hispanica.

### Data availability.

This genome sequence has been deposited in GenBank with the accession number MW557853. The associated BioProject, SRA, and BioSample accession numbers are PRJNA698715, SRR13683896, and SAMN17734765, respectively.
